# Reconsideration of Nanowire Growth Theory at Low Temperatures

**DOI:** 10.3390/nano11092378

**Published:** 2021-09-13

**Authors:** Vladimir G. Dubrovskii

**Affiliations:** Faculty of Physics, St. Petersburg State University, Universitetskaya Emb. 13B, 199034 St. Petersburg, Russia; dubrovskii@mail.ioffe.ru

**Keywords:** semiconductor nanowires, growth rate, length, radius, surface diffusion, modeling

## Abstract

We present a growth model that describes the nanowire length and radius versus time in the absence of evaporation or scattering of semiconductor atoms (group III atoms in the case of III-V NWs) from the substrate, nanowire sidewalls or catalyst nanoparticle. The model applies equally well to low-temperature metal-catalyzed or selective area growth of elemental or III-V nanowires on patterned substrates. Surface diffusion transport and radial growth on the nanowire sidewalls are carefully considered under the constraint of the total material balance, yielding some new effects. The nanowire growth process is shown to proceed in two steps. In the first step, the nanowire length increases linearly with time and is inversely proportional to the nanowire radius squared and the nanowire surface density, without radial growth. In the second step, the nanowire length obeys the Chini equation, resulting in a non-linear increase in length with time and radial growth. The nanowire radii converge to a stationary value in the large time limit, showing a kind of size-narrowing effect. The model fits the data on the growth kinetics of a single self-catalyzed GaAs nanowire on a Si substrate well.

## 1. Introduction

Semiconductor nanowires (NWs), particularly III-V NWs, are widely considered as fundamental building blocks for nano-research and are useful for applications in nanoelectronics and nanophotonics [1]. These NWs are produced by different epitaxy techniques, including molecular beam epitaxy (MBE) and vapor phase epitaxy (VPE) via the metal-catalyzed vapor-liquid-solid (VLS) growth (with either Au [2] or group III [3] droplets) or catalyst-free selective area growth (SAG) [4]. The VLS growth and SAG can even coexist in one epitaxial process depending on the NW radius and the growth conditions [5]. At a low enough temperature for a given NW material, Au-catalyzed VLS growth is transitioned to the vapor-solid-solid (VSS) one, where a catalyst nanoparticle becomes solid [6]. Progress in synthesis, characterization and device functionalization of semiconductor NWs has been closely associated with the growth modeling (see, for example, Ref. [7] for a review of the early results and Ref. [8] for a review of recent studies). In many cases, the NW growth features determine their morphology, crystal phase and statistical properties [8].

In self-catalyzed VLS growth of III-V NWs, the axial NW growth rate is given by the group V input to the droplet [9]. However, the Ga balance has recently been considered to model the GaP NW morphology evolution in the arrays [10]. In Au-catalyzed VLS growth of different III-V NWs, the axial NW growth rate, dL/dt, is usually assumed to be group III limited, while the radial growth is simply ignored in most models [11,12,13,14,15,16,17,18,19,20,21]. Group III adatoms can diffuse to the NW top from the NW sidewalls and/or the substrate surface, yielding the $R^{-1}$ [11,12,13,14] or $R^{-2}$ [15,16] radius dependence of dL/dt. If adatoms are collected from the entire NW length  L, the NW length increases exponentially with time. This exponential growth has been discussed since the early stages of NW research [7] but was observed much later for Au-catalyzed InP_1−x_As_x_ NWs grown by MBE on unpatterned substrates [17] and Au-catalyzed InAs NWs grown by metal-organic VPE on patterned substrates [18]. The influence of group V atoms on the NW growth rate, ignored in the earlier models [11,12,13,14,15,16], may result in the suppression of the nucleation rate on the NW top facet [19,20,21,22]. According to the current view [19,20,21,22], the axial growth of III-V NWs can be limited by either surface diffusion of group III adatoms or incorporation (nucleation) on the NW top, depending on the NW radius and growth conditions used. The $R^{-2}$ dependence of the NW axial growth rate, controlled by surface diffusion from the substrate, has been experimentally observed for MBE-grown, Au-catalyzed GaAs [15] and InP [23] NWs. 

The radial NW growth occurs in most NWs regardless of the growth method and is due to a limited diffusivity of the growth species on the NW sidewalls. It has been thoroughly studied, both experimentally and theoretically, for Au-catalyzed [24] and self-catalyzed [10,25] VLS III-V NWs as well as for non-VLS III-V NWs obtained by SAG [4,26,27]. The group III adatom sink considered in the corresponding diffusion equations and resulting in a finite diffusion length on the NW sidewalls [11,12,13,14,15,16,17,18,19,20,21,22,24,27] can be caused by either desorption or surface incorporation, leading to radial growth in the latter case. In the directional MBE deposition method, the neighboring NWs are influenced by a severe shadowing effect [10]. On the other hand, re-evaporated (or scattered) atoms may land on the lateral facets or catalyst nanoparticles of the growing NWs [10,28,29,30]. This collective effect of material exchange between the NWs and the substrate surface influences the NW growth rates and morphology. At low enough temperatures, however, re-evaporation of the material should largely decrease, while the surface diffusion remains as the main kinetic pathway for the material exchange. 

Consequently, here we develop the NW growth theory in the absence of desorption of a semiconductor (Si or Ge) or group III (Ga or In) atoms from the substrate, NW sidewalls or catalyst nanoparticles, using the material balance for these atoms and considering their surface diffusion that contributes into the axial and radial growths. As in earlier works [13,14,15,16,17,18,19,20,21,22], we consider the diffusion of material directly deposited on the nanowire sidewalls, adatom diffusion from the substrate surface up along the NW and material deposited directly on the NW top (onto a metal particle in the VLS growth mode). There are, however, two important differences: (i) All the deposited material in our model must remain in the NWs or on the substrate surface, and (ii) the limited diffusion length on the NW sidewalls is due to surface incorporation and must contribute to the radial growth. The model applies to VLS, SAG or VSS growth of NWs on patterned substrates [30] or, more generally, to low-temperature NW growth proceeding concomitantly with the formation of a parasitic two-dimensional layer (2D) or surface islands between the NWs [31]. By definition, the low-temperature NW growth considered here requires that no material is evaporated or scattered from different surfaces and that a finite diffusion length on the NW sidewalls is limited by surface evaporation causing the radial NW growth. Whenever these conditions apply, the model can also be used for growth modeling of II-VI, oxide or III-N NWs. 

## 2. Model

**Figure 1:**
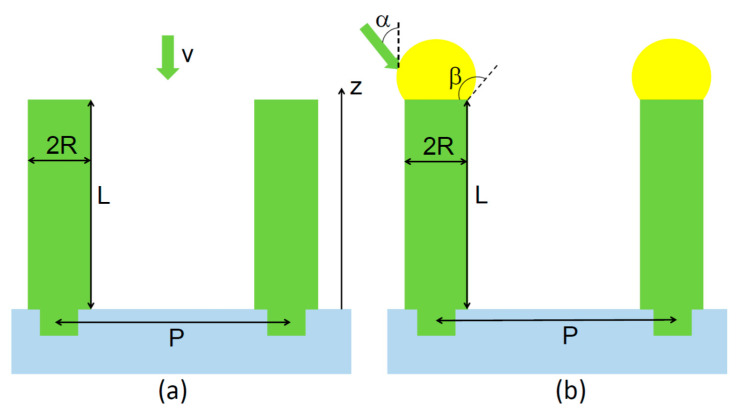
Illustration of the model geometry in (**a**) SAG and (**b**) VLS growth with a metallic droplet on top.

The model geometry is illustrated in Figure 1 for SAG or metal-catalyzed NW growth. Assuming for simplicity that cylindrical NWs have the same length L above the surface (and excluding the height of a catalyst nanoparticle) and the radius R, the material balance in the absence of desorption writes;

(1) $$H=vt=\pi R^{2}LN$$

where v=JΩ is the effective deposition rate in nm/s (with J as the corresponding atomic flux of semiconductor material or group III atoms and Ω as the elementary volume in solid), t is the NW growth time (starting from the moment where the NWs emerge from the substrate surface), H is the effective deposition thickness and N is the surface density of NWs related to the pitch P shown in Figure 1. If a fraction of the deposited material remains in the parasitic 2D layer or islands, $H_{0}$ is the total deposition thickness and $H_{2D}$ is the thickness of the 2D layer, with the corresponding growth rates $v_{0}$ and $v_{2D}$ (the latter is assumed time-independent). Then the effective deposition rate becomes $v=v_{0}-v_{2D}$. In this way, we account for a limited diffusion length of adatoms on the substrate surface, leading to 2D growth at a rate of $v_{2D}$. When $v_{2D}=0$, the surface diffusion length is much larger than the distance between the NWs, and all the adatoms deposited onto the substrate are able to diffuse to the NW base. More complex scenarios with a time-dependent $v_{2D}$ (for example, due to the shadowing effect in MBE) will be studied elsewhere. 

From Equation (1) we have

(2) $$\pi \frac{d\left(R^{2}L\right)}{dt}=\frac{v}{N},$$

meaning that the derivative of the NW volume with respect to time is a constant that is inversely proportional to the NW density. At a time-independent NW radius $R=R_{0}$, determined, for example, by the size of pinholes in an oxide layer, the aspect ratio $L/H=1/\pi R_{0}^{2}N$ gives the magnifying effect of NW growth with respect to 2D growth, which can reach very high values and increases for lower NW density and smaller radius. 

The axial NW growt rate is generally given by [11,12,13,14,15,16,17]

(3) $$\frac{dL}{dt}=\chi v+{\left(\frac{dL}{dt}\right)}_{diff},\;{\left(\frac{dL}{dt}\right)}_{diff}=-\frac{2\Omega D}{R}{\left(\frac{dn}{dz}\right)}_{z=L}$$

here χ=2/(1+cosβ) in VLS VPE where the vapor phase surrounds the droplet, and χ=1 in SAG (at β=0), with  β as the droplet contact angle as shown in Figure 1. In the directional MBE method, the vapor flux on the apical droplet depends on the contact angle β and the beam angle α, as discussed in Ref. [32]. Considering the low-temperature growth, we ignore any possible contribution from re-emitted species and include the diffusion-induced contribution ${\left(dL/dt\right)}_{diff}$, with n as the adatom surface density on the NW sidewalls, which depends on the vertical coordinate z, and D as the diffusion coefficient of adatoms on the NW sidewalls. Therefore, surface diffusion (from the NW sidewalls and the substrate) is the only source of additional material supply to NWs, as in Ref. [33].

The steady-state diffusion equation on the NW sidewalls, written here in the case of VPE, is given by [11,12,13,14,15].

(4) $$D\frac{d^{2}n}{dz^{2}}+J-\frac{n}{\tau }=0$$

where the linear sink −n/τ can only be due to the radial growth. The adsorbing boundary condition at the NW top

(5) n(z=L)=0

excludes any possible downward diffusion flux away from the catalyst nanoparticle [12,21]. The second boundary condition at the NW base should now be obtained from the material balance

(6) $$\chi J\pi R^{2}+2\pi RLJ-2\pi RD{\left(\frac{dn}{dz}\right)}_{z=0}=\frac{\pi }{\Omega }\frac{d\left(R^{2}L\right)}{dt},$$

which is different from the previous works [11,12,13,14,15,16,17]. Here, all the material fluxes arriving onto the NW through its top (the first term), the NW sidewalls (the second term) and surface diffusion from the substrate (the third term) contribute to the change of the NW volume. Using Equation (6), the boundary condition at the NW base is obtained in the form

(7) $$-{\left(\frac{dn}{dz}\right)}_{z=0}=\frac{v}{\Omega }\left(\frac{1}{N}-\chi \pi R^{2}-2\pi RL\right)$$

The solution to Equation (4) with the boundary conditions given by Equations (5) and (7) is

(8) $$n\left(z\right)=J\tau -Csinh\left(z/\lambda \right)+\frac{Csinh\left(L/\lambda \right)-J\tau }{cosh\left(L/\lambda \right)}\cos h\left(z/\lambda \right),$$

where $\lambda =\sqrt{D\tau }$ is the effective diffusion length of sidewalls adatoms (limited by the surface incorporation and radial growth on the NW sidewalls), and

(9) $$C=\frac{v\lambda }{2\pi RD\Omega }\left(\frac{1}{N}-\chi \pi R^{2}-2\pi RL\right)$$

Calculating the diffusion-induced axial growth rate and summing it up with the direct flux as given by Equation (3), we arrive at

(10) $$\frac{dL}{dt}=v\left[\chi +\frac{2\lambda \varphi }{R}tanh\left(L/\lambda \right)+\left(\frac{1}{\pi R^{2}N}-\chi -\frac{2L\varphi }{R}\right)\frac{1}{cosh\left(L/\lambda \right)}\right]$$

This expression also applies to the case of MBE, with χ given in Ref. [32] and φ=tanα/π as the geometrical factor of MBE growth [10,21]. The first bracket term in Equation (10) gives the contribution from the direct flux, the second term gives the contribution of the adatom diffusion to the NW top from its side facets, while the third term stands for surface diffusion from the substrate. According to Equation (1), the NW radius is related to its length as

(11) $$R={\left(\frac{vt}{\pi LN}\right)}^{1/2}$$

at any time. The shadowing effect, which is not directly included in these considerations, may change the growth kinetics of long-enough NWs in MBE. This interesting question will be studied elsewhere. 

## 3. Results and Discussion

Short NWs with L/λ≪1 grow only axially at a time-independent radius

(12) $$L=\frac{vt}{\pi R_{0}^{2}N}=\frac{H}{\pi R_{0}^{2}N},\;R=R_{0},$$

because no surface incorporation occurs in this step. This is the simplest form of the growth equation in the absence of desorption [31,34] showing that each NW receives a portion of the material flux, which is inversely proportional to the NW density and the NW radius squared. No exponential growth stage is observed, and no direct impingement term is present, because the adatoms collected by the NW sidewalls and top (with or without a catalyst nanoparticle) are lost in exactly the same amount on the substrate surface. In MBE, this corresponds to the shadowing effect of a single NW on the adatom collection area on the substrate. A similar equation (without any loss of material on the substrate surface at $H_{2D}=0$) was earlier discussed in connection with synergetic NW growth [34] and was used as the upper limit for the NW length corresponding to the maximum possible axial growth rate. 

Using Equation (10) at L/λ≫1 for dL/dt and Equation (11) for R, long NWs with L/λ≫1  evolve according to

(13) $$\begin{array}{cc} \frac{dL}{dH}=\chi +\varepsilon {\left(\frac{L}{H}\right)}^{1/2} & ,\;L\left(H=H_{*}\right)=L_{*}\\ \;\;\;\;\varepsilon =2\lambda \varphi {\left(\pi N\right)}^{1/2} \end{array}$$

where the initial condition is set at the effective thickness $H_{*}$ after which the approximation L/λ≫1 can be applied without losing accuracy, starting from the NW length $L_{*}$. Comparing it to the previously used axial growth rate of the form dL/dH=χ+2λφ/R at a time-independent $R=R_{0}$ (Refs. [13,14,15,17,18,19]) we can see how important the difference is between the sidewall diffusion length limited by desorption (yielding $R=R_{0}$ and leaving only parametric dependence of the axial growth rate on the NW radius) amd by surface incorporation (leading to radial growth and a time-dependent NW radius which reduces the axial NW growth rate). 

Equation (13) is a special type of the Chini equation, which is integrated by using the substitution of the unknown function U=L/H, yielding dL=UdH+HdU and reducing Equation (13) to the separable differential equation $-dH/H=dU/(U-\varepsilon \sqrt{U}-\chi )$. Upon integrating, we get

(14) $$H=H_{*}\frac{F\left(L/H\right)}{F\left(L_{*}/H_{*}\right)},\;F\left(L/H\right)=\frac{1}{L/H-\varepsilon \sqrt{L/H}-\chi }{\left(\frac{2\sqrt{L/H}+\sqrt{\varepsilon^{2}+4\chi }-\varepsilon }{2\sqrt{L/H}-\sqrt{\varepsilon^{2}+4\chi }-\varepsilon }\right)}^{\frac{\varepsilon }{\sqrt{\varepsilon^{2}+4\chi }}}.$$

Asymptotic behavior of the solutions at large H→∞ is given by

(15) $$\frac{L}{H}\to \frac{1}{4}{\left(\sqrt{\varepsilon^{2}+4\chi }+\varepsilon \right)}^{2},\;\frac{R}{R_{N}}\to \frac{2}{\sqrt{\varepsilon^{2}+4\chi }+\varepsilon },$$

where $R_{N}=1/\sqrt{\pi N}$ is the spacing (average distance) between the NWs. This behavior is quite interesting and shows that the NWs will never merge ($R<R_{N}$) provided that χ>1 even at ε→0, in which case χ gives the magnifying effect of the nanoparticle surface in the VLS or VSS growth mode. In SAG at χ=1, the NWs can remain higher than the deposition thickness only due to surface diffusion (ε>1). The stationary NW radius is quite large and far exceeds the initial NW radius $R_{0}$.

**Figure 2:**
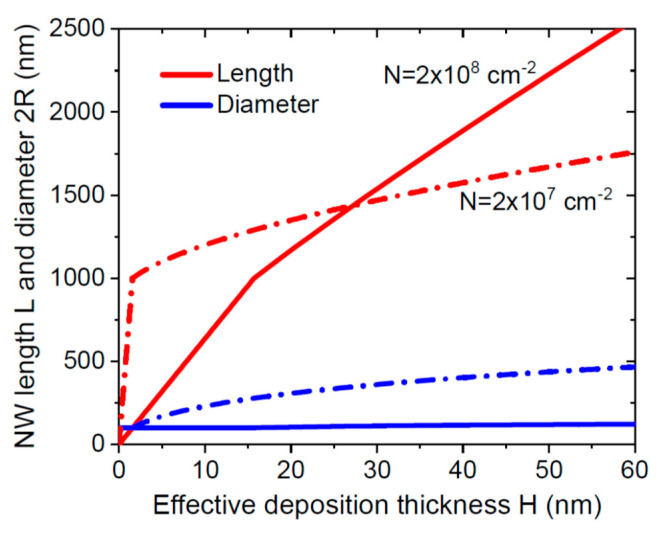
NW length and diameter versus H  at a fixed λ of 1000 nm and $R_{0}=$ 50 nm, for two different surface densities N indicated in the legends. The curves are obtained in the case of VPE SAG (χ=φ=1 ). The rapid axial NW growth at a constant radius is converged to the slower one at L=λ. Lower-density NWs grow faster in the first step but slower towards the end. The effective deposition thickness is related to the growth time as H=vt, so that the curves can equivalently be presented as functions of t. The same applies to the curves shown in Figure 3.

Furthermore, the parameter ε scales with the NW density as $N^{1/2}$ and hence decreases for lower surface densities. As a consequence, the NWs grow slower for lower N, while they grow faster for lower N in the first step (at $R=R_{0}$). This feature is illustrated in Figure 2, showing the simplified form of solutions at $\varepsilon \sqrt{L/H}\gg \chi$,

(16) $$L≅{\left[\sqrt{L_{*}}+\varepsilon \left(\sqrt{H}-\sqrt{H_{*}}\right)\right]}^{2},$$

and R given by Equation (11). Here, we also use the “intuitive” approximation of $L_{*}=\lambda$, as in Ref. [18], assuming a sharp transition between the two growth regimes after the NW length reaches the diffusion length of group III adatoms. With λ= 1000 nm and $R_{0}=$ 50 nm, the main model parameters ${\left(\pi R_{0}^{2}N\right)}^{-1}$ and ε equal 63.7 and 5.01 at N= 2 × 10^8^ cm^−2^, changing to 637 and 1.59 at N= 2 × 10^7^ cm^−2^.

**Figure 3:**
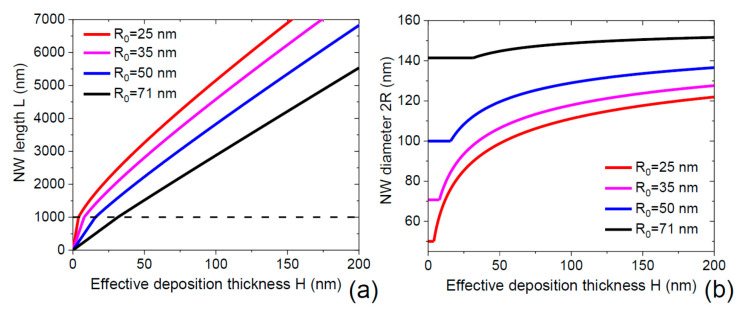
NW length (**a**) and diameter (**b**) versus H  at a fixed λ of 1000 nm (indicated by the dashed line in (**a**)) and N= 2 × 10^8^ cm^−2^, for different initial NW radii $R_{0}$ indicated in the legends. The NW lengths increase linearly with H below 1000 nm and sub-linearly above 1000 nm. The NW diameters stay constant below 1000 nm length and increase above 1000 nm length, converging to the same stationary diameter at H→∞. This focusing effect is expected to narrow the diameter distribution within an ensemble of NWs having different initial diameters.

Figure 3 show the evolution of the NW length and diameter for different initial NW radii $R_{0}$ from 25 nm to 71 nm, at a fixed N= 2 × 10^8^ cm^−2^ and other parameters as in Figure 2. The NW lengths increase linearly before reaching 1000 nm, at the rates that increase for smaller $R_{0}$. Above 1000 nm, the NW lengths increase sub-linearly due to the radial growth. 

It is interesting to note that the NW radii, which are very different in the first growth step, converge to the same stationary radius in the asymptotic stage (at H→∞), corresponding to a narrowing effect on the radius distribution of NWs if one would start with a distribution of the NW radii at the beginning of growth. This effect has been known in the specific case of self-catalyzed III-V NWs [35,36]. Here, the size narrowing is caused simply by the fact that the radius of initially thinner NWs grows faster compared to thicker NWs, as seen in Figure 3b. Of course, our simplified model considers an idealized array of identical NWs, in which case the radius self-stabilization means that the NWs grown in differently sized pinholes will all have the same radius after a long enough growth time. We suspect, however, that a similar effect should pertain in an ensemble of NWs with different initial radii.

The radial growth above the critical NW length quite significantly changes the diffusion-like dependence of the NW lengths on the NW radii measured after a given deposition time or deposition thickness. Therefore, the earlier models, which ignore the radial growth and yield of the $R^{-1}$ or $R^{-2}$ radius correlation of the NW length [11,12,13,14,15,16,17,18,19,20,21,22,23], should be re-considered from the viewpoint of the results obtained here. 

**Figure 4:**
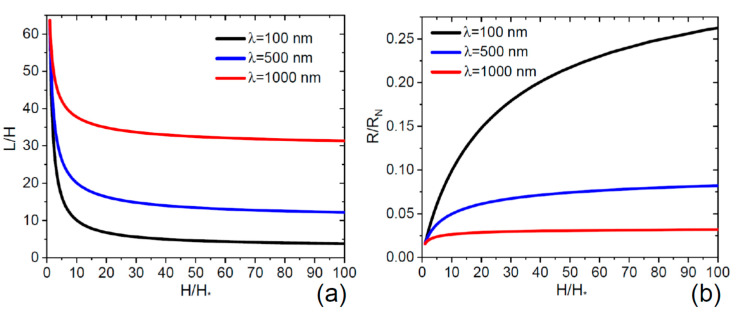
Aspect ratios L/H (**a**) and $R/R_{N}$ (**b**) versus the normalized deposition thickness $H/H_{*}$, obtained from the general solutions at a fixed $R_{0}=$ 50 nm and N= 2 × 10^8^ cm^−2^ for different diffusion lengths λ indicated in the legends. The ratio L/H  in (**a**) decreases with the deposition thickness from its initial value of 63.7 to a stationary value that is higher for a larger diffusion length. Larger λ lead to lower rates of radial growth and smaller stationary NW radii in (**b**).

Figure 4 show the aspect ratios L/H and $R/R_{N}$ versus $H/H_{*}$, where $H_{*}$ is the deposition thickness at which the NW length equals the diffusion length of group III adatoms. These curves are obtained from the general expressions given by Equations (11) and (14) for VPE growth at β=90° (χ=2, φ=1), at a fixed $R_{0}=$50 nm, N= 2 × 10^8^ cm^−2^ (corresponding to a constant $\lambda /H_{*}=$63.7) and different diffusion lengths λ from 100 nm to 1000 nm. The ratio L/H in the second growth step decreases to a stationary value, which is higher for longer λ, while the ratio $R/R_{N}$ increases to a stationary value, which is lower for longer λ. As expected, higher diffusivity of adatoms on the NW sidewalls yields longer and thinner NWs. With a short diffusion length of 100 nm, the stationary ratio L/H decreases from a high initial value of 63.7 to only 4, whereas the NWs becomes more than 10 times thicker than at the beginning. 

**Figure 5:**
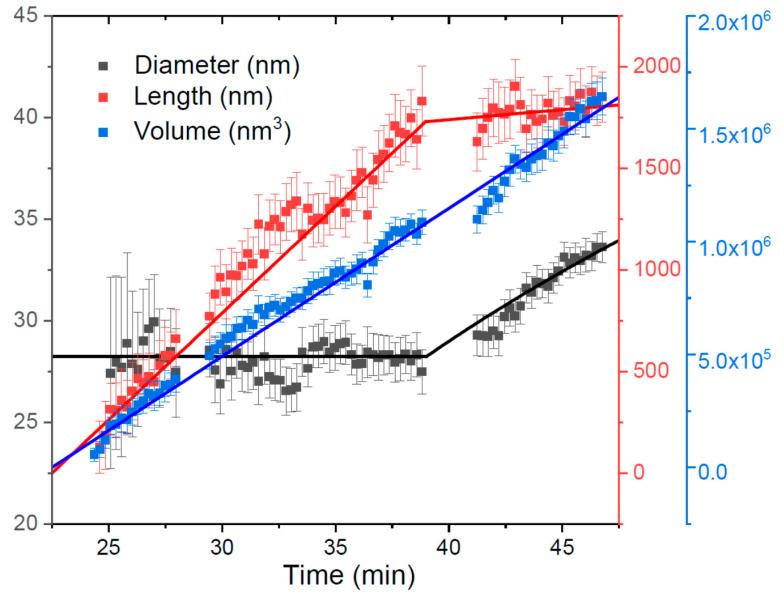
Diameter, length and volume of a single self-catalyzed GaAs NW grown by MBE on patterned Si(111) substrate. Symbols show the data obtained by in situ XRD analysis [30]; lines are the fits obtained within the model.

Figure 5 shows the data on the growth kinetics of a single self-catalyzed GaAs NW obtained by an in situ micro-X-ray diffraction (μXRD) technique in Ref. [30]. GaAs NWs were grown using a portable MBE mounted onto the diffractometer at a beamline of Petra III in DESY, in patterned arrays of pinholes in SiO_x_/Si(111) with Ga pre-deposition, at a temperature of 610 °C and with an equivalent 2D deposition rate of 2.1 nm/min. The NWs grew concomitantly with parasitic GaAs nano-islands that formed unintentionally on the oxide mask surface. The diameter and volume of an individual GaAs NW were recorded in situ by μXRD, after which the length was calculated assuming s cylindrical NW geometry. The NWs and islands started to form after an incubation time of 22.5 min. The NW volume V  evolved linearly with time, with a constant growth rate of 65,600 nm^3^/min. The NW radius stayed constant at ~14 nm until the NW length reached the diffusion length of Ga adatoms on the NW sidewalls of ~1730 nm. These numbers yield a magnifying factor L/H of 50 in the first growth step. After that, the NW length is well fitted by Equation (16) at $L_{*}=\lambda =$1730 nm, $H_{*}=$34.6 nm, and ε= 1.0. It is seen that the radial growth in the second step takes most of the deposited GaAs, leading to severe suppression of the axial growth. Similar behavior was observed earlier and modelled in Reference [24] for Au-catalyzed GaAs NWs grown by MBE, with a close diffusion length of 1500 nm.

## 4. Conclusions

In conclusion, we have developed a generalization of the growth theory for low-temperature NW growth in MBE, VPE or SAG techniques. In the absence of evaporation or scattering of semiconductor (group III) atoms, the only source of additional material supply to NWs is the surface diffusion transport, while the sidewall adatoms, which do not reach the NW top, contribute to the radial growth on the NW sidewalls. Due to the total material balance, the entire growth process proceeds in two steps. In the first step, NWs grow only axially, with the length being inversely proportional to the surface density and the NW radius squared. In the second step, the NW length and radius increase non-linearly with time or deposition thickness. The length of lower-density NWs grows slower in the second step, while it grows faster in the first step. The NW radius increases to a stationary value, which is independent of the initial radius, leading to a narrowing effect on the radius distribution. In the asymptotic regime (at large H), the NW length is greater than H due to the magnifying effect of a catalyst nanoparticle in metal-catalyzed growth, but mainly due to surface diffusion from the NW sidewalls to the top. The model fits the data on the growth kinetic of a single self-catalyzed GaAs NW quite well and can be further used for modeling and optimizing the NW morphology in a wide range of material systems for deposition techniques where surface diffusion constitutes the main kinetic pathway for both axial and radial NW growth.
